# Human Neural Stem Cells Flown into Space Proliferate and Generate Young Neurons

**DOI:** 10.3390/app9194042

**Published:** 2019-09-27

**Authors:** Carlos Cepeda, Laurent Vergnes, Nicholas Carpo, Matthew J. Schibler, Laurent A. Bentolila, Fathi Karouia, Araceli Espinosa-Jeffrey

**Affiliations:** 1 Departments of Psychiatry, UCLA, Los Angeles, CA 90095, USA;; 2 Department of Chemistry and Biochemistry, University of California, Los Angeles, CA 90095, USA;; 3 Advanced Light Microscopy/Spectroscopy, California NanoSystems Institute, University of California, Los Angeles, CA 90095, USA; 4 Department of Pharmaceutical Chemistry, University of California San Francisco, San Francisco, CA 94158, USA;; 5 NASA Ames Research Center, Space Biosciences Research Branch, Moffett Field, CA 94035, USA

**Keywords:** neural stem cells, pluripotency, space flight, microgravity, proliferation, neuronal specification, neurons, energetics, glycolysis

## Abstract

Here we demonstrate that human neural stem cells (NSCs) proliferate while in space and they express specific NSC markers after being in space. NSCs displayed both higher oxygen consumption and glycolysis than ground controls. These cells also kept their ability to become young neurons. Electrophysiological recordings of space NSC-derived neurons showed immature cell membrane properties characterized by small capacitance and very high input resistance. Current injections elicited only an incipient action potential. No spontaneous synaptic events could be detected, suggesting their immature status even though most recorded cells displayed complex morphology and numerous cell processes. Ascertaining the origin of the NSCs′ increased energy requirement is of the essence in order to design effective measures to minimize health risks associated with long-duration human spaceflight missions.

## 1. Introduction

The long-term effects of the space environment on the central nervous system (CNS) still remain largely unknown. Human space travelers experience a unique environment that affects homeostasis and physiological adaptation. In particular, neuro-psycho-physiological health deserves special attention to ensure successful long-term space missions. In recent years, studies have started to address neural function and behavior in space. It has been reported that cerebellar, sensorimotor, and vestibular brain areas seem to be the most affected [1]. Microgravity reduces central venous and intracranial pressure; nonetheless, intracranial pressure is not reduced to the levels observed in the 90° degrees seated upright posture on Earth. Instead, the basal levels over 24 h in microgravity pressure in the brain are slightly higher than those on Earth, which may cause the remodeling of the eye in astronauts [2]. Moreover, a space flight-associated neuro-ocular syndrome (SANS) has been recently described [3]. At the cellular level, it has been shown that microgravity induces apoptosis, alters the cytoskeleton, and affects the differentiation, proliferation, and metabolic status in different cells [4]. Nonetheless, only a few aspects regarding the sensitivity of human neural stem cells (hNSCs) in microgravity have been reported. An interesting article from Silvano and collaborators [5] showed that murine cerebellar neural stem cells (NSCs) respond to simulated microgravity by the arrest of their cell cycle in S-phase and enhanced apoptosis. They also found that these changes were transient and upon return to normal gravity (1 G) these cells recovered their stemness and a normal cell cycle.

The objective of the present study was to determine how the space environment influences human-induced pluripotent stem cell-derived NSCs, their biology, and their fate choice capability toward the neuronal phenotype.

## 2. Methodological Approach

NSCs were derived from induced pluripotent stem cells. The original cells, known as “CS83iCTR-33nxx” (such as skin cells or lymphoblasts), were “reprogrammed” and provided to us by the Cedars-Sinai Medical Center via a material transfer agreement.

NSCs were seeded onto an 8-well Petri dish from Airbus-Kiwi (Friedrichshafen, DE), flown to the International Space Station (ISS), and installed in the Space Technology and Advanced Research System Experiment Facility-1 at 37 °C. The cells remained onboard the ISS for 39.3 days and then returned to Earth.

After splashdown, transport to Long Beach airport, and delivery to UCLA, the NSCs were retrieved from the hardware, plated onto poly-d-lysine-coated flasks in our proprietary stem cell chemically defined medium (STM) [6,7], and allowed to recover from space flight. Subsequently, some of these NSCs were plated in neuronal specification medium (NSM).

## 3. Results

### 3.1. NSCs in Space Environment (SPC) Preserve Their “Stemness” and Proliferate

**Scheme 1.**
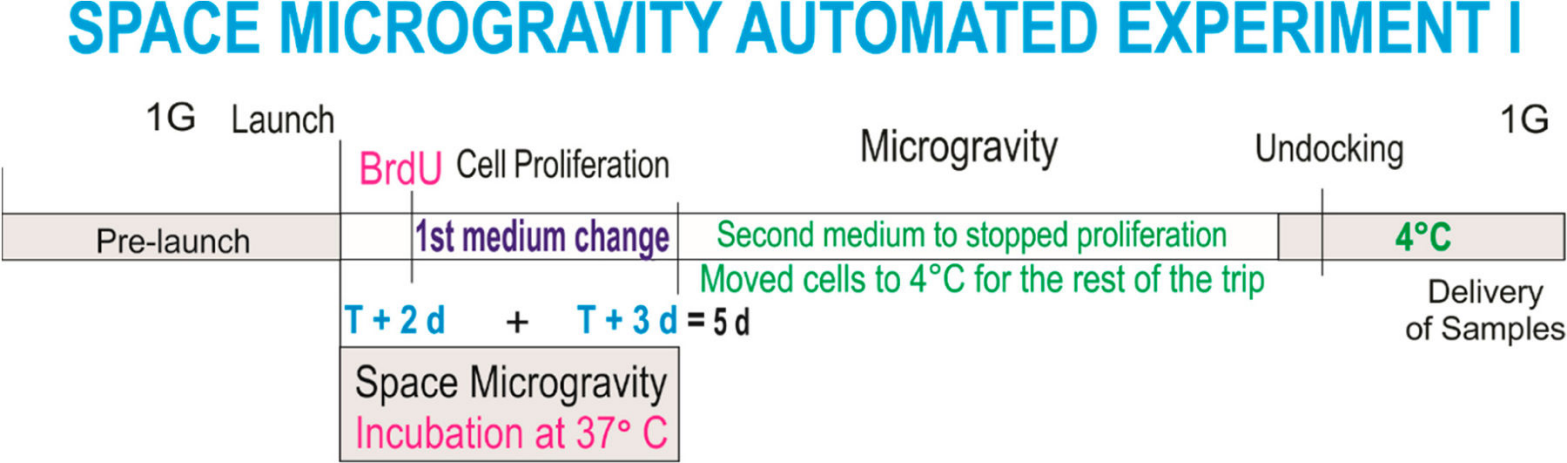
Timeline 1: This diagram illustrates the times at which culture media changes took place while neural stem cells (NSCs) traveled and were in space in the automated hardware at 37 °C. Culture medium from Tank 1 containing bromodeoxyuridine (BrdU) was released into the cell chamber 2 days after docking. Three days later (at 5 days of having been in space), the second medium without BrdU was released and the units were stored at 4 °C. Proliferation while in space was ascertained during only 3 days.

This experiment was designed to ascertain the proliferation of NSCs solely while in microgravity. In order to elucidate if NSCs in “space microgravity” proliferate, we used the Type IV automated experiment insert from Kiwi (Airbus, Friedrichshafen, DE), allowing for a fresh medium change twice during space travel. The medium within which the cells traveled was replaced with fresh medium containing bromodeoxyuridine (BrdU) two days after the cargo berth to the ISS. Therefore, BrdU incorporation took place during three days. NSC cultures were then arrested with the second stem cell medium and placed at 4 °C for further examination upon return to the laboratory (Scheme 1).

**Figure 1.**
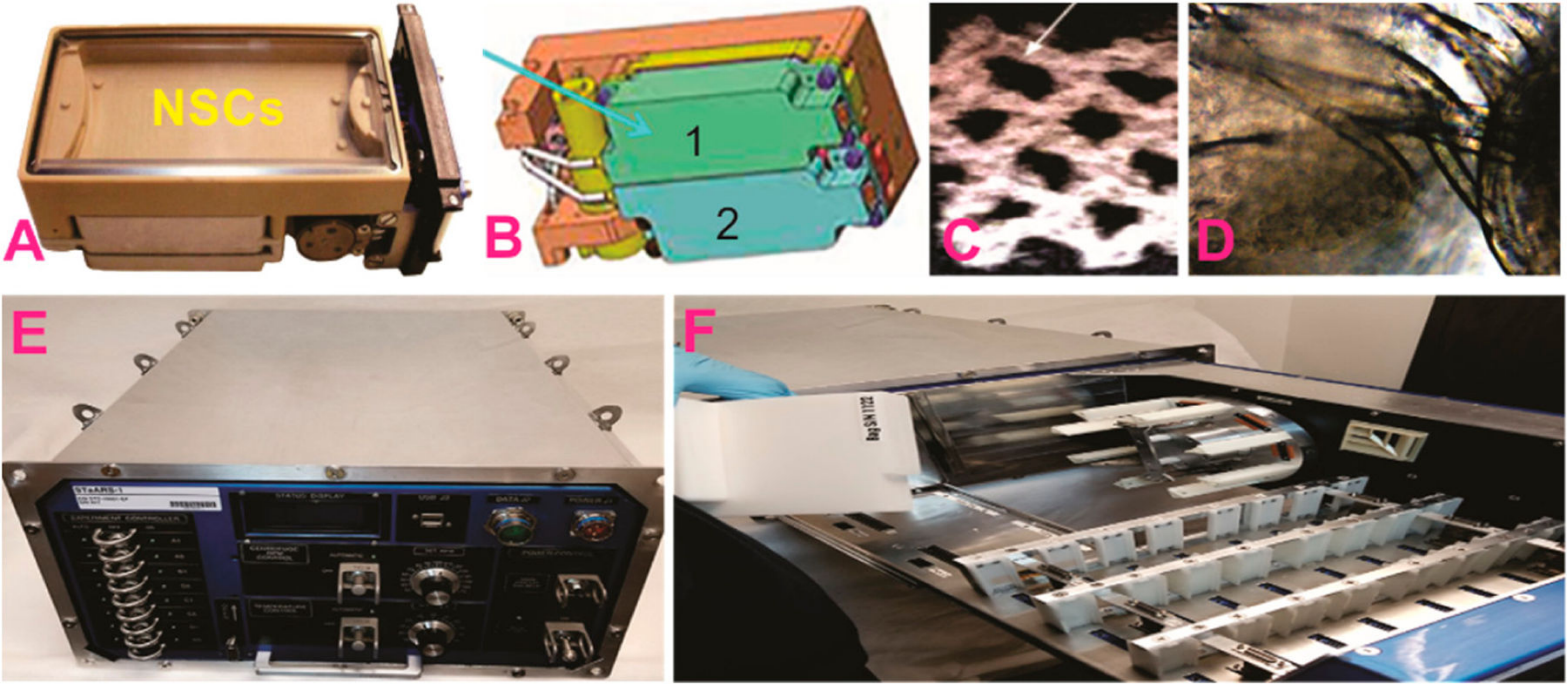
(**A**) View of an automated Type IV Unit Kiwi (Airbus). (**B**) Tank 1 (green) contained NSCs, medium, and BrdU. At time (T) + 2 this medium was delivered to NSCs, at T + 5 medium from the second tank (blue) was delivered to arrest the cells and the units were placed at 4 °C. Two units containing 12 pieces of mesh carrier with cells were used for the “in space” proliferation study (**C**) Representative view of the mesh carrier on which tire NSCs were seeded. (**D**) Mesh with NSCs recovered from the devices post-flight. (**E,F**) Views of StaARS-1, which offers real-time command capability without the need for astronaut time.

NSCs were seeded onto a mesh-cell carrier because cells adhere firmly to it and have a better resistance to vibrations and g-forces during take-off and re-entry to the atmosphere. This protocol was tested and verified during the science definition phase of the investigation following NASA’s requirements (Figure 1A).

**Figure 2.**
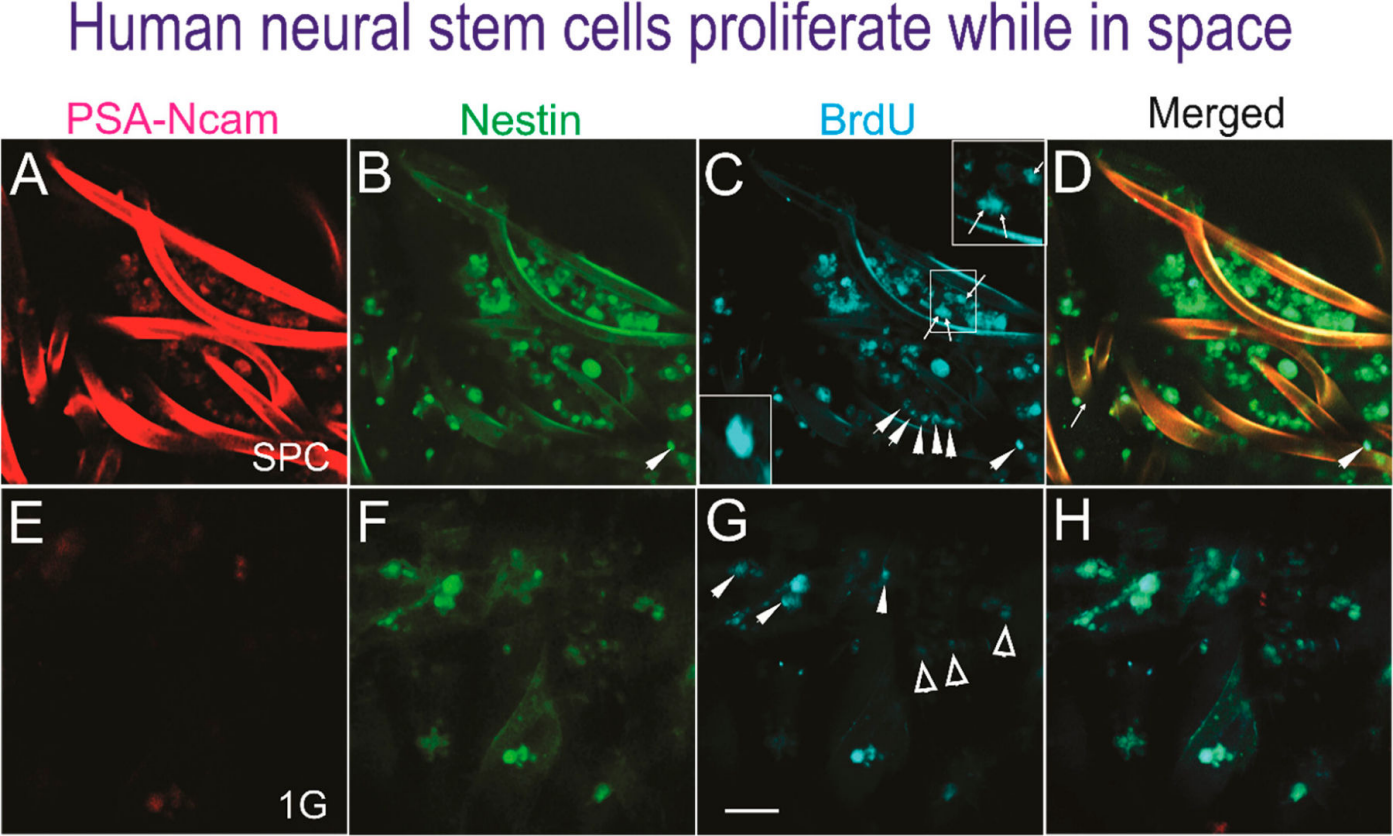
Human NSCs proliferate in space. Triple immunofluorescence performed on cells adhered to the mesh-cell carrier. A to D show NSCs that proliferated while in space. E to H show views of ground control NSCs. (**A**) The PSA-NCAM antibody labeled most but not all cells. (**B**) Cells expressing nestin as seen by the intensity of the marker in all NSCs. (**C**) Different intensities of BrdU-labeled cells, some organized in rows (arrowheads) or as dividing cells (arrows) as shown in the insets. (**D**) Most cells expressed BrdU and nestin and a subpopulation colocalized with PSA-NCAM. (**E**) PSA-NCAM was not expressed by the NSCr grown in 1 G (ground control). (**F**) Some cells expressed nestin at different intensities and others were negative. (**G**) Only some cells intensely expressed BrdU (arrowheads), while most had a faint expression (open arrowheads) or were negative. (**H**) The merged image shows that there were less cells in 1 G (ground controls) with respect to the cells sent to space. The BrdU incubation was for 72 h for all cells in automated units. (Bar = 50 μm).

Upon return, the NSCs were recovered and post-fixed to perform triple immunofluorescence against two NSC markers as previously described [6]—polysialic acid (PSA) cell adhesion molecule (NCAM), known as PSA-NCAM, and the intermediate filament protein nestin, also known as a neural stem/progenitor cell marker—as well as BrdU for the detection of new DNA as a marker for cell proliferation (Figure 2).

### 3.2. Passive Experiments

**Figure 3.**
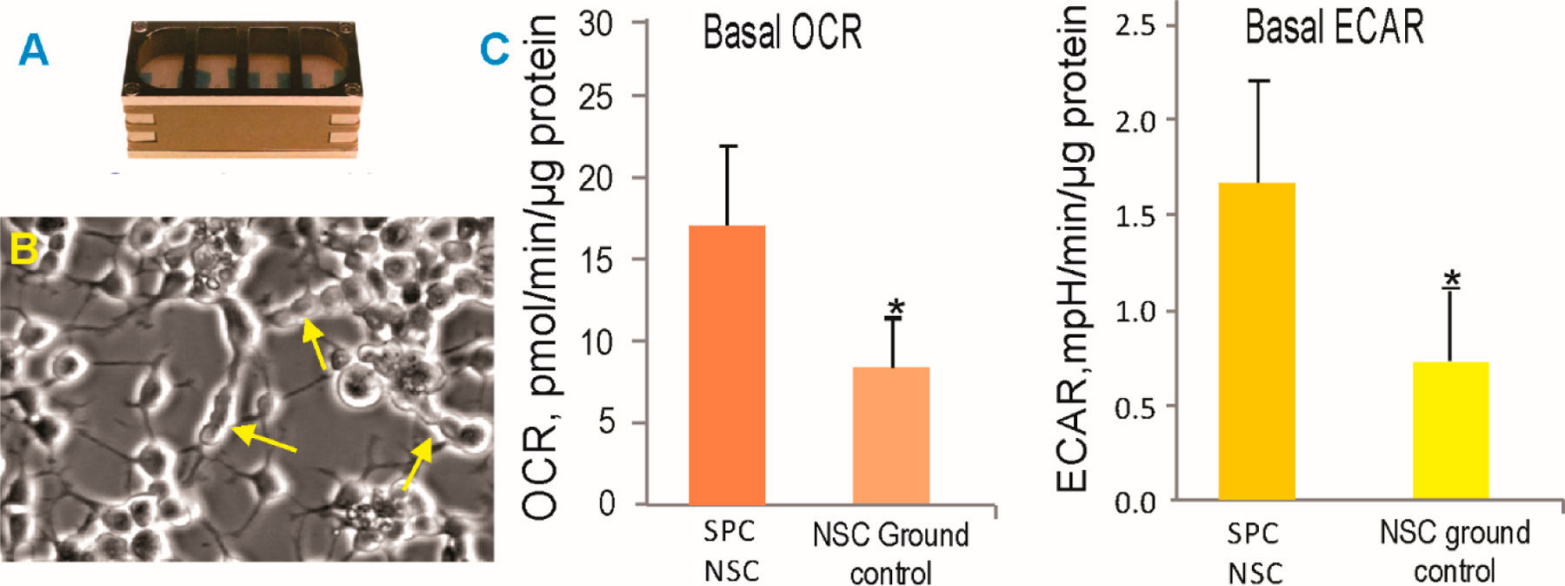
SPC-NSCs and ground control NSCs were recovered from the passive hardware (**A**) and treated in the same manner. (**B**) Representative example of past-flight NSCs that continued to actively proliferate, as shown by the arrows. (**C**) After 39.3 days in space, NSCs display enhanced energy consumption. OCR—oxygen consumption rate; ECAR—extracellular acidification rete; SPC-NSCs in the space environment. Each experiment consisted of n = 12. The values are expressed as mean + SD. * The Student’s t test revealed that these differences were significant (p < 0.05).

In order to ascertain the health status of the NSCs, we performed experiments using 8-well Petri dishes from Kiwi (Airbus). We have called these passive experiments, because tire cells remained within the same culture medium for 45 days (counting from handover to handover), having spent a total of 39.3 days in space from launch to splashdown of the dragon vehicle and the rest of the time in transit. Upon return, the cells were harvested from the hardware and seeded on poly-d-lysine. After the recovery period, the NSCs were seeded in Seahorse Bioscience XFplates (Agilent) to assess their cellular bioenergetic status. Tire oxygen consumption rare (OCR) and the extracellular acidification rate (ECAR, a measure of glycolysis) were both higher in the NSCs exposed to the space environment as compared to the ground control cells, indicating a higher metabolic state (Figure 3). These measurements were performed one week after the NSCs returned to Earth. Therefore, one can infer that NSCs “remember” having been in microgravity. Alternatively, re-adaptation to 1 G may have prompted higher energetic needs.

### 3.3. Fate Choice

**Scheme 2.**
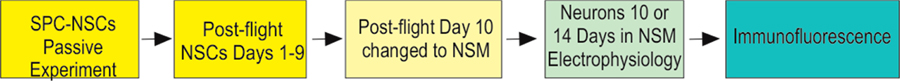
Timeline 2.

After the recovery period, the NSCs were fed with neuronal specification medium (NSM) [6] in order to obtain neurons. Exposure to a space environment did not alter the NSCs potential to choose the neuronal fate. Interestingly, neuroblasts proliferated for more than 1 week after being in NSM before moving toward maturation. The timeline for neuronal specification is shown in Scheme 2.

#### Neuronal Specification.

Upon their return to Earth, the cells were allowed to recover in NSC medium and standard conditions for 9 days. NSCs were introduced to neuronal specification medium (NSM) on Day 10. After 10 or 14 days of having been in NSM, we examined the electrophysiological properties of these cells.

**Figure 4.**
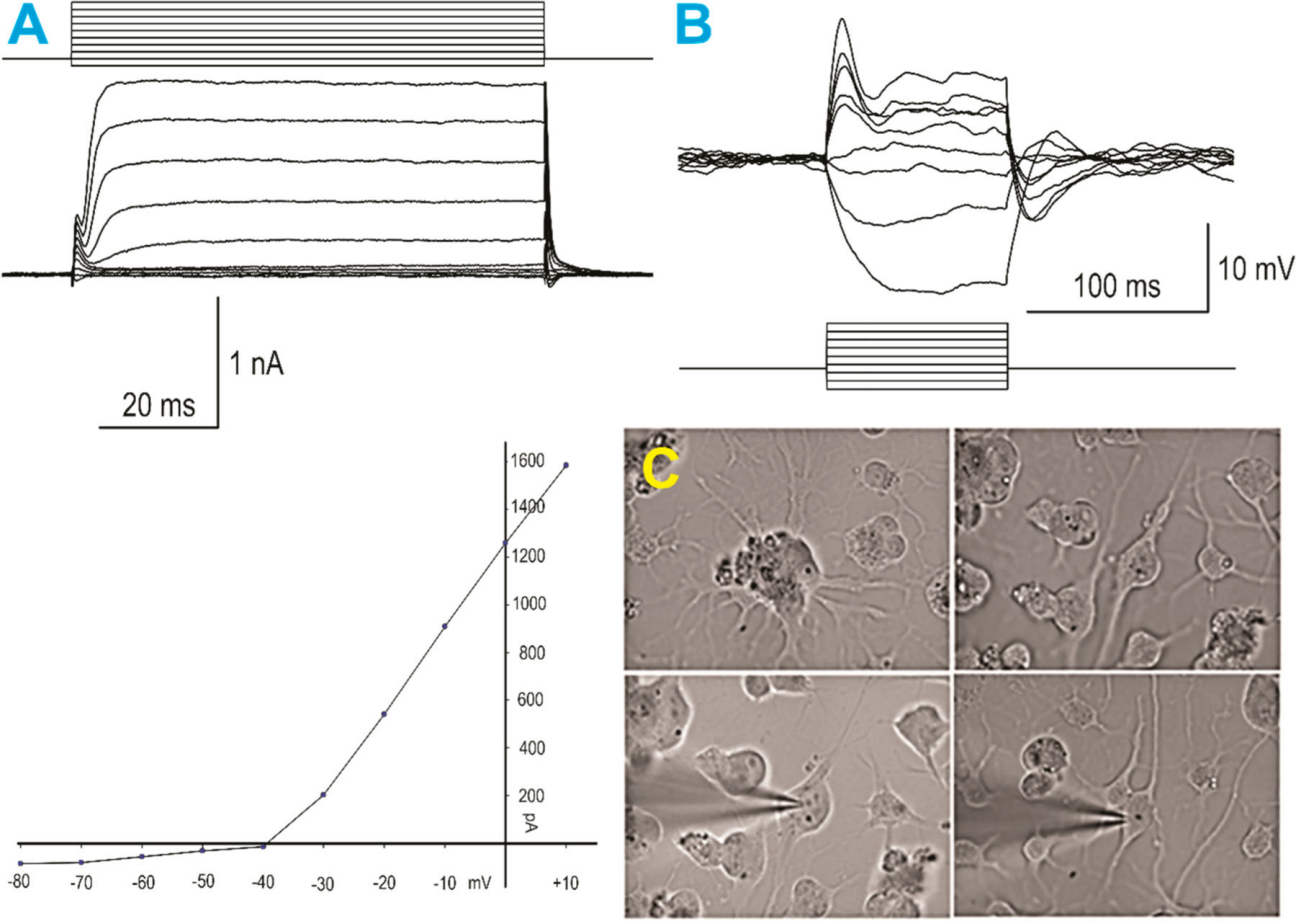
SPC-NSCs gave rise to neurons after space flight. (**A**)—(left panels) Whole-cell voltage clamp recording; using; K-gluconate in the internal solution: (top) step voltage commands from −80 to +10 mV elicited only minimal inward currents, whereas outward currents were predominant, and (bottom) graph represents an IV relationship, illustrating the presence of large outward currents starting at −30 mV. (**B**) When the recording was switched to current clamp mode, small step current pulses evoked large negative voltage deflections and only an incipient action potential with depolarizing current injections, demonstrating a neuronal phenotype, yet still very immature. (**C**) Infrared video-microscopy images of human neurons derived from NSCs flown into space. The top left panel shows a cluster of cells with multiple processes emanating from different cells. The top right panel shows an NSC with bipolar morphology. The bottom left panel shows the same cell after patch and electrophysiological recordings (illustrated in A and B) were obtained. The bottom right panel shows another NSC after patching.

Electrophysiological recordings of SPC-NSC-derived neurons were obtained 10 days or 14 days after being in NSM using standard procedures, as detailed in our previous publication [8] Figure 4.

A total of seven plates containing SPC-NSCs were used, and 16 cells were recorded in voltage and current clamp modes using a K-gluconate-based internal solution. First, passive membrane properties were determined in the voltage clamp mode. The cell membrane capacitance was relatively small (27.3 ± 1.9 pF, mean ± SEM), while the input resistance was high (553.4 ± 84.7 MΩ). The decay time constant was very fast (0.4 ± 0.05 ms). These properties are similar to those obtained from implanted NSCs [8]. After measuring passive membrane properties, the recording was switched to the current clamp mode. The mean resting membrane potential was relatively depolarized (−38.1 ± 1.8 mV), and upon injection of depolarizing current steps, most cells displayed a small-amplitude action potential, suggesting that these SPC-NSCs were in an immature state. In the gap free mode, we determined that spontaneous synaptic activity was not yet apparent.

## 4. Discussion

Here we have demonstrated that human NSCs proliferate while in space and that they remain as NSCs after 39.3 days unattended, in the same culture medium, and in space microgravity. Moreover, these cells also keep their ability to become young neurons in the appropriate conditions. Silvano’s team reported data supporting that simulated microgravity arrests cell proliferation, albeit in a transient manner, and they also found that these changes were not permanent as NSCs recovered their proliferative capacity when they were returned to normal gravity.

The apparent contradiction between Silvano’s group reporting the arrest of proliferation date and this study may reside in the fact that the cells they used were from a different species (i.e., mouse vs. human). Additionally, their cells were kept in simulated microgravity far 3 to 5 days, as opposed to our NSCs, which were exposed to SPC microgravity tor 5 days at 37 °C and in BrdU for 3 days. Therefore, the reasons for the apparent contradiction may be due to the following: (1) the extent of the exposure to microgravity, which might be a determinant for the arrest of cell proliferation in Silvano’s study or (2) perhaps simulated microgravity differs from space microgravity with respect to the regulation of neural cells and their biological processes. Why a higher bioenergetic state is elicited by microgravity is not clear at the present time. The fact that we demonstrated that NSCs proliferate in space needs to be studied in more detail, as it might be that this phenomenon contributes to the visual impairment and intracranial pressure syndrome (VIIP) exhibited by astronauts in space and upon their return to Earth [9]. Elucidating the origin of the increased energy needs by brain cells is crucial in order to design effective countermeasures for astronauts working onboard the International Space Station and for long-term space travel. Moreover, here we found that NSCs displayed an elevated energy metabolism; both oxygen consumption and glycolysis were higher than in the ground control counterparts, confirming our previous findings from work using “simulated microgravity”[10]. We have previously reported that simulated microgravity enhances mitochondrial function in oligodendrocytes (OLs), the myelin forming cells in the CNS [11]. Two of the main sources of energy for respiration and energy production, glucose and pyruvate, were decreased after just 3 days in microgravity, while lactate levels increased considerably indicating that OLs switched to anaerobic glycolysis in order to meet the energy demands elicited by microgravity [11].

It is possible that NSCs in space require more energy for proliferation as well as for maintaining homeostasis in an environment where, besides microgravity, there are other factors involved, such as radiation, moving while being packed and unpacked, and variation of g-forces occurring during launching and splashdown. Nonetheless, the cells appeared to be able to recover from such a journey and display their stemness when returned to Earth. Furthermore, as mentioned above, their membrane properties were similar to the NSCs grown in normal gravity.

## 5. Conclusions

Neurobiology and the environment determine psychological and physiological fitness and behavior; hence, the maintenance of energy metabolism homeostasis is of paramount importance. The formulation of energy rich diets for astronauts, as well as providing an enriched environment, may contribute to reaching and maintaining the balance we all strive for—mens sana in corpore sano (a healthy mind in a healthy body).

The results presented in this communication are also promising for humankind on Earth; neurodegenerative diseases frequently result from the loss of a specific cell population, and it was demonstrated that microgravity is an excellent strategy to increase neural cell numbers without the need of performing genetic manipulations or long-term treatments with mitogens. It is believed that incorporating microgravity into projects of a translational nature will lead the scientific community one step closer toward translational research to address neurodegeneration and the recovery of CNS function.

## Featured Application:

Understanding how neural stem cells behave in the microgravity environment in space will help us develop suitable countermeasures to protect astronauts from problems with intracranial hypertension during and after long-term space travel. Studying the effects of microgravity on the regulation of neural cell proliferation and their commitment to a specific lineage (i.e., neurons) will offer benefits to patients on Earth for cell replacement therapies using self-donor cells to address neurodegenerative and developmental disorders and diseases, such as cerebral palsy or multiple sclerosis.
